# Mission accomplished?

**DOI:** 10.1007/s10143-021-01608-z

**Published:** 2021-08-16

**Authors:** Ulrich Sure

**Affiliations:** Department of Neurosurgery and Spine Surgery, University Hospital Essen, Essen, Germany

Dear highly esteemed readership and editorial team of *Neurosurgical Review*,

When I took over responsibility as Editor-in-Chief of *Neurosurgical Review* in 2016, the journal went through a number of difficult, but necessary, changes. After the journal’s remarkable success story under the editorship of Helmut Bertalanffy, who had worked to achieve this success, Springer Nature, and more precisely Springer-Verlag GmbH, changed the format of the journal from print to exclusively online. By also changing the managing editorial software, the editorial team introduced a number of new and important features, such as a plagiarism check for any manuscript submitted since 2017. In the beginning, my personal aim was to further promote the already high quality of the journal and guide it to becoming the journal with the highest impact factor among all the European neurosurgical journals.

To do so, I needed considerable support from local collaborators, and I therefore built up a highly motivated editorial team in Essen. In the past 5 years, this editorial team consisted of numerous important “helping hands and minds.” Most recently, the following colleagues have contributed to the success of *Neurosurgical Review*: Editorial Office, Sandra Braun; and Editorial Assistants (in alphabetical order), PD Dr. Y. Ahmadipour, Dr. L. Barthel, Dr. B Chen, PD Dr. M. Darkwah Oppong, Dr. O. Gembruch, Dr. S. Hetze, PD Dr. R. Jabbarli, PD. Dr. D. Pierscianek, and Dr. L. Rauschenbach. I am most grateful to all these people for their commitment and excellent work. In addition, Springer Verlag GmbH has supported my efforts to improve the quality of *Neurosurgical Review* by providing a highly qualified and motivated managing team at the publisher end; I would particularly like to highlight the merits of Dr. M. F. Bartels.

With the support of all these people, *Neurosurgical Review* succeeded in dramatically increasing the number of submitted manuscripts (nearly 4-fold), the number of published Online First articles (2-fold), and the article downloads (2-fold) (see Table 1). These important objectives were achieved thanks to a short and effective review process for all manuscripts submitted.

**Table 1:** Numbers for *Neurosurgical Review* 01/2016–06/2021

Year	2016	2017	2018	2019	2020	2021
Submissions	260	347	437	584	956	484 (until 30/06/2021)
Accepted Articles	56	128	133	155	234	129 (until 30/06/2021)
Articles published Online First	121	129	125	153	225	137 (until 30/06/2021)
Downloads	105,152	112,660	109,332	131,962	195,194	132830 (until 31/05/2021)
Impact Factor	**2.060**	**2.255**	**2.532**	**2.654**	**3.042**	-

Case reports and poor-quality papers were rejected immediately, without peer review in many cases. Unfortunately, this means that the overall rejection rate also increased. However, a large number of high-quality manuscripts still remained for the peer review process. Therefore, we are extremely grateful to all our reviewers and the entire international editorial board, since all these people used their expertise to help us to identify the best papers among all manuscripts submitted for publication in *Neurosurgical Review*. The research for these papers originated on all continents, making the journal even more international than before.

As a consequence, I feel that the quality of papers accepted for publication has further increased over the past 5 years. This impression was recently confirmed by the increase in our impact factor (2020) to 3,042: an all-time high in the history of *Neurosurgical Review* (see Table 1).

So, in summary, after a demanding and strenuous 5 years as editor, I would like to declare:

*My mission is accomplished!*

However, I believe that our originally European, and increasingly international journal, *Neurosurgical Review,* has an even greater potential for further development. I hope that our readership, as well as all our contributors, agrees with me when I say:

*Yes, the mission is accomplished, but not yet finished!*

Thank you all once again for your constant, and hopefully continued, support!

Ulrich Sure,

Essen, Germany

